# A Health Informatics System in the South Australian Public Health Network: Implementation Report

**DOI:** 10.2196/86564

**Published:** 2026-09-01

**Authors:** James Malycha, Jake White, Adam Phillips, Lukah Dykes, Lukasz Wiklendt, Paul Lambert, Adelaide Boylan, Justin Beilby

**Affiliations:** 1 Central Adelaide Local Health Network, The Queen Elizabeth Hospital Intensive Care Unit, 28 Woodville Rd, Woodville South, Adelaide, 5011, Australia, 61 0419004939; 2 Adelaide Medical School, Faculty of Health and Medical Sciences, Adelaide University, Adelaide, Australia; 3 Clinical and Health Sciences, University of South Australia, Adelaide, Australia; 4 Torrens University Australia, Adelaide, Australia

**Keywords:** health informatics, governance, electronic medical record, implementation report, AI, health information system

## Abstract

**Background:**

Following 2 decades of electronic medical record (EMR) adoption, most large public health systems hold comprehensive digital clinical data but lack the complementary informatics capability to return those data to clinicians, coders, and operational teams in a usable form. In South Australia (SA), the statewide Sunrise (Altera Digital Health) EMR system has digitized documentation and ordering since 2017; however, tools for back-end data extraction and enriched clinical information displays were not prioritized, and clinical, operational, and research users have reported ongoing difficulties accessing timely data.

**Objective:**

The aim of this study is to describe the development, governance, and deployment of a cloud-native health informatics system (HIS) in the Central Adelaide Local Health Network (CALHN), which serves approximately 40% of SA public patients, and to report implementation outcomes in accordance with the iCHECK-DH (Guidelines and Checklist for the Reporting on Digital Health Implementations) guidelines.

**Methods:**

The HIS comprises 6 architectural layers deployed on Microsoft Azure with Red Hat OpenShift (IBM), extracting Sunrise EMR data in near real time under a read-only model in which all patient data remain within the SA Health network at all times. Implementation proceeded through 5 overlapping phases (2021-2026), governed across 4 domains: technical (security impact assessment, information asset classification, and independent cybersecurity review), clinical (a clinical governance committee that has met quarterly since May 2022), corporate (incorporation of HeartAI Pty Ltd in 2022, with conflicts of interest declared and managed under SA Health policy), and ethical (human research ethics committee [HREC], reference 18079 with subsequent amendments). Development was funded through approximately Aus $1.84 million (Aus $1=US $0.72 as of August 24, 2026) in competitive and institutional grants under a public-private model with CALHN and AusHealth.

**Implementation (Results):**

Three applications have reached deliberately different stages of maturity. The CALHN Critical Care Informatics System (CCCIS) has been implemented and evaluated: it has been deployed across the 2 CALHN intensive care units (ICUs; 54 beds, approximately 5000 admissions per year) since 2022, is in daily clinical use, and automates the submission of 115 variables to the Australian and New Zealand Intensive Care Society Centre for Outcome and Resource Evaluation (ANZICS CORE) registry. In a formal evaluation by 8 senior intensivists, its mean System Usability Scale score was 76 of 100, and the overall workload was low (NASA Task Load Index: 21/100). CODEXA, an AI-assisted clinical coding application, is in operational validation: models trained on 500,000 episodes and tested on 50,000 held-out episodes achieved a pooled *F*_1_-score of 71% across the full case mix and 57.86% (precision 64.72%; recall 55.53%) on complex acute episodes, in line with published benchmarks of 58% to 61% obtained from curated research datasets. The Patient Flow application is in co-design with CALHN’s Network Operations Centre, with interface prototype evaluations completed in July 2026. In May 2026, the platform received unconditional endorsement from Digital Health SA’s Technical Design Review Committee, the highest technical governance approval in SA Health, concluding a 5-year governance pathway.

**Conclusions:**

A locally developed informatics platform can be governed, deployed, and validated in a public health system; demonstrating clinical outcomes and transitioning to operational status are the next priorities.

## Introduction

### Context and Problem

This implementation report describes the development and deployment of a health informatics system (HIS) in the Central Adelaide Local Health Network (CALHN) in South Australia (SA), which includes the Royal Adelaide Hospital and The Queen Elizabeth Hospital and services approximately 40% of SA public patients. The report is in accordance with the iCHECK-DH (Guidelines and Checklist for the Reporting on Digital Health Implementations) guidelines and checklist (Checklist 1) [1]. The implementation took place in the context of Australia’s National Digital Health Strategy (2023‐2028), which prioritizes interoperability and secondary use of clinical data, and SA Health’s digital transformation agenda, which consolidated electronic medical record (EMR) infrastructure; however, complementary analytics and decision-support capabilities remained underdeveloped.

In 2011, SA public hospitals began migrating from paper-based records to the Sunrise (Altera Digital Health) EMR system. The implementation was a major public digital infrastructure project and was successful in the context of its resource and time constraints. Tools enabling back-end data extraction and enriched clinical information displays were not prioritized. Clinical staff subsequently reported frustration with the usability of clinical displays, and operational and research teams reported difficulty extracting timely data for reporting and analysis (World Health Organization Classification of Digital Health Interventions, items 2.1.1, 2.3.1, 2.5, and 2.10) [2].

### The Intervention

Three sequential projects were funded and implemented within CALHN, each delivering a digital tool for a specific user group: critical care clinicians (real-time decision support at the bedside), clinical coders (coding accuracy and hospital funding allocation), and operations managers (bed availability and patient flow).

The 3 projects are at deliberately different stages of maturity, and this distinction is maintained throughout this report: the CALHN Critical Care Informatics System (CCCIS) has been implemented and evaluated; CODEXA, the clinical coding application, is in operational validation; and the Patient Flow application is in co-design. Throughout this report, “HIS” refers to the platform; CCCIS, CODEXA, and Patient Flow are the applications built upon the platform; and HeartAI Pty Ltd is the company involved in the platform’s development and commercialization.

### Similar Interventions

Most large health networks in high-income countries have completed EMR migrations and are at varying stages of maturity in the secondary use of clinical data [3,4]. This implementation was primarily inspired by the Ambient Warning and Response Evaluation (AWARE) intensive care unit (ICU) information system at the Mayo Clinic, a clinician-facing system that aggregates EMR data into structured dashboards to support bedside decision-making, which has been evaluated in randomized crossover trials [5,6]. The HIS shares AWARE’s approach, which restructures raw EMR data into clinician-centered displays, but differs in architecture (cloud-native, open-standard technologies rather than proprietary infrastructure) and scope (extending beyond critical care to coding and operational flow). An extended comparison with other international and commercial systems is provided in Multimedia Appendix 1.

## Methods

### Aims, Objectives, and Evaluation Framework

The primary aim of this project was to develop and deploy a contemporary HIS in SA public hospitals, built with technology capable of extension across SA Health. Table 1 summarizes the platform’s objectives, their measurable indicators, the corresponding governance records, and their status.

**Table 1.** HIS evaluation framework.

Objective	Measurable indicators	Governance record	Status as of September 2026
A digital platform capable of supporting an informatics system in the SA^a^ Health system	Platform deployed on Microsoft Azure with OpenShift (IBM) orchestration; infrastructure-as-code repository established; independent cybersecurity review passed	Azure resource deployment logs; Terraform version-control repository; SIA^b^ documentation; external cybersecurity audit report	Achieved; cloud-native platform operational since 2022 with 6 architectural layers deployed and maintained under version control
Governance structures to enable platform connection to the EMR^c^ system	SIA initiated and maintained; IAC^d^ completed; Clinical Governance Committee established and meeting quarterly	SIA records (initiated 2021, ongoing); IAC completion certificate (2021); Clinical Governance Committee minutes (quarterly from May 2022)	Achieved; all required governance structures in place: SIA ongoing, IAC completed, Clinical Governance Committee operational with quarterly meetings since May 2022
Platform connection to the EMR system	Live data extraction from Sunrise (Altera Digital Health) EMR back end established; DHSA^e^ formal approval obtained	DHSA approval documentation; system connectivity logs; EMR data extraction validation records	Achieved; EMR integration established in May 2022 with formal DHSA approval; live data extraction operational across CCCIS^f^ and CODEXA
Supported projects that reflect the platform’s functionality in areas of need in the health service	Number of funded projects deployed; user groups served; usability evaluations conducted; HREC^g^ approvals obtained	Grant agreements and milestone reports; HREC approval records (ref. 18079, 17287); usability study protocols and findings (see companion paper)	Partially achieved; 3 projects funded and at explicitly different stages: CCCIS implemented and evaluated (2 ICUs^h^); CODEXA in operational validation; Patient Flow in co-design
Commercial agreements that support platform development and deployment	Number of executed agreements; total competitive grant funding secured; commercial ownership structures formalized	Executed agreements between HeartAI Pty Ltd, CALHN, and AusHealth; ASIC^i^ shareholder registry; grant award letters	Achieved: 4 grant-funded agreements totaling approximately Aus $1.84 million (2022‐2026); public-private ownership model operational with joint IP^j^ arrangements for each project

a SA: South Australia.

b SIA: security impact assessment.

c EMR: electronic medical record.

d IAC: information asset classification.

e DHSA: Digital Health South Australia.

f CCCIS: CALHN (Central Adelaide Local Health Network) Critical Care Informatics System.

g HREC: human research ethics committee.

h ICU: intensive care unit.

i ASIC: Australian Securities and Investments Commission.

j IP: intellectual property.

### Platform Architecture and Data Governance

**Figure 1.**
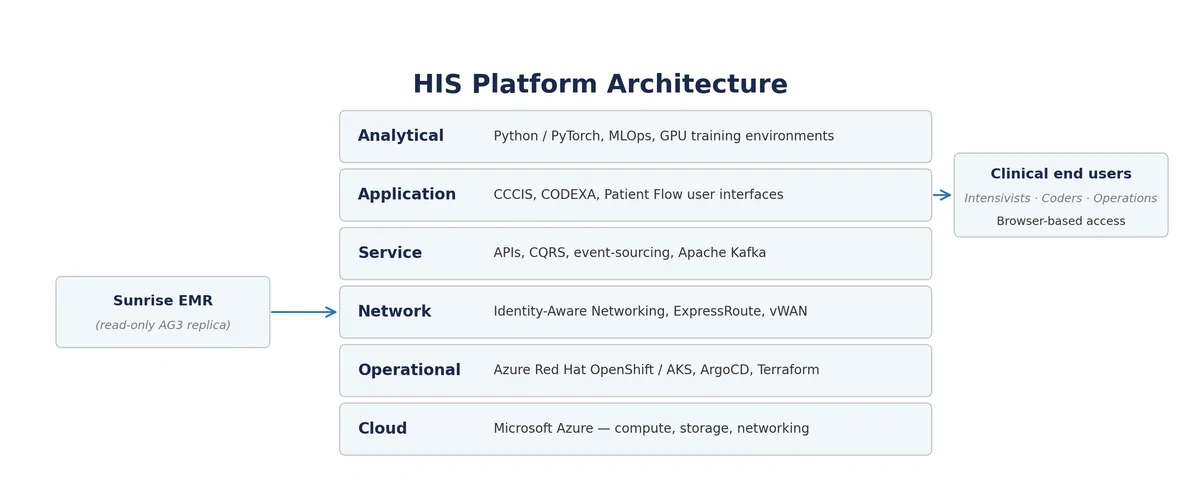
Health informatics system (HIS) platform architecture. AKS: Azure Kubernetes Service; CCCIS: CALHN (Central Adelaide Local Health Network) Critical Care Informatics System; CQRS: command query responsibility segregation; GPU: graphics processing unit; MLOps: machine learning operations; vWAN: virtual wide area network.

The HIS comprises 6 architectural layers (Figure 1). In the cloud layer, Microsoft Azure provides computing, storage, and networking managed through a version-controlled, infrastructure-as-code approach [7]. The operational layer uses Red Hat OpenShift (IBM) for container orchestration [8]. The network layer enforces access controls that keep clinical data within defined security boundaries. The service layer manages interapplication communication and near–real-time processing of EMR data, which is essential for clinical displays that must reflect current patient status. The application layer hosts CCCIS, CODEXA, and Patient Flow, and the analytical layer supports AI model training within restricted, graphics processing unit–enabled environments. Extended technical details, including the deployment tooling and software patterns used in each layer, are provided in Multimedia Appendix 1.

Data access is controlled through an identity management framework that assigns users to groups and roles on a minimum-necessary basis, with encryption at rest and in transit, auditing of modifications, and detection of unusual access patterns. All patient data remain within the SA Health network at all times. The HIS accesses EMR data under the governance and security frameworks of SA Health and CALHN, and no patient data are held by or transferred to HeartAI Pty Ltd or any external entity. Data sovereignty therefore remains with the public health system regardless of the commercial structures supporting the platform’s development.

### Governance

In SA, EMRs are owned and governed by the SA government through the Department for Health and Wellbeing, SA Health, and Digital Health SA (DHSA), which manages information technology across the health system. Integration of the HIS with the Sunrise EMR system (achieved May 2022) required formal approvals across 4 governance domains, described in brief here and in full in Multimedia Appendix 1.

Technical governance comprised a security impact assessment (initiated in 2021 and ongoing), an information asset classification (completed in 2021), and an independent external cybersecurity review (completed in 2022). On May 6, 2026, the DHSA Technical Design Review Committee (TDRC) unconditionally endorsed the HIS platform deployment. TDRC endorsement is the highest formal step in SA Health technical governance, and this concluded a 5-year process from initial security review to design baseline approval.

Clinical governance is provided by the Clinical Governance Committee, which has met quarterly since May 2022. Membership includes the CALHN chief executive officer (or delegate), the executive lead for critical care and perioperative medicine, the director of digital strategy, a consumer representative, and senior critical care clinicians, with dedicated project subcommittees reporting to the Clinical Governance Committee.

### Ethical Considerations

Corporate governance was established through the incorporation of HeartAI Pty Ltd in February 2022, enabling formal, legally binding partnerships with SA Health, DHSA, CALHN, and AusHealth. Ethics approval for CCCIS was granted by the CALHN human research ethics committee (HREC) in 2023 (reference 18079), with amendments extending coverage to CODEXA (reference 17287) and the Patient Flow project (pending review). This approval governs the research activities conducted using the HIS; the platform’s technical development preceded the research program and was governed by the technical processes above.

The involvement of a private company in the development and commercialization of a platform serving a public health system creates actual and perceived conflicts of interest. All authors except JB hold equity in HeartAI Pty Ltd, and these conflicts have been declared through the appropriate institutional pathways at CALHN. Four mechanisms manage them: independent oversight by the Clinical Governance Committee, whose membership includes non–equity holders; joint ownership of the products developed under the CCCIS agreement by CALHN and AusHealth rather than by HeartAI alone; commercialization through AusHealth, a self-funded medical research charity that reinvests revenue into health innovation; and research priorities set by publicly funded grant agreements rather than by HeartAI’s commercial interests.

### Funding and Commercial Model

Early platform builds, implementation frameworks, and project protocols were completed on personal time before incorporation. Four grant-funded agreements followed between December 2022 and April 2026, totaling approximately Aus $1.84 million across CCCIS, CODEXA, and Patient Flow (Table 2). Private equity from 11 registered shareholders has supported core platform development and corporate operations, comprising a minority of total funding; its value is not disclosed for commercial confidentiality reasons.

**Table 2.** Summary of budget and funding sources (2022-2026), totaling approximately Aus $1.84 million.

Funding source/mechanism	Project supported	Funding amount (Aus $)	Duration	Funding body/agreement	Notes
CALHN^a^ Innovation Award	CALHN CCCIS^b^	200,000	2 years (2022‐2024)	CALHN Internal Innovation Grant	Initial grant enabling first HIS^c^ deployment
AusHealth Collaborative Research and Commercialisation Agreement	CCCIS expansion and platform integration	312,000	2 years (2023‐2025)	AusHealth	Enabled transition to operational maturity
AusHealth Collaborative Research and Commercialisation Agreement	Clinical coding software (CODEXA version 1)	770,000	2 years (2024‐2026)	AusHealth	Funded AI-assisted coding solution
AusHealth Collaborative Research and Commercialisation Agreement	CODEXA version 2	360,000	7 months (2026)	AusHealth	Commercially ready AI-assisted coding MVP^d^
CALHN/AusHealth joint funding	Patient Flow	200,000	1 year (2025‐2026)	CALHN and AusHealth	Joint project extending HIS capability
Private equity and in-kind contributions	HeartAI core platform and corporate operations	Undisclosed (multiple tranches)	Ongoing since 2022	11 registered shareholders	Equity investment and in-kind contributions

a CALHN: Central Adelaide Local Health Network.

b CCCIS: CALHN Critical Care Informatics System.

c HIS: health informatics system.

d MVP: minimum viable product.

Under commercial agreements, CCCIS and Patient Flow are jointly owned by CALHN and AusHealth, so the South Australian public remains a direct beneficiary, while AusHealth retains ownership of CODEXA with a share of revenue directed to HeartAI Pty Ltd. Sustainability planning couples this hybrid funding with governance informed by published frameworks for AI governance and ethics in health care [9-12], a transition path from research-only to operational funding through CALHN’s digital health budget, and progressive Fast Healthcare Interoperability Resources adoption. Milestone-based grant funding has at times constrained project continuity and resourcing, and the concentration of funding across a small number of grant bodies creates dependency risk. Extended funding and sustainability details are provided in Multimedia Appendix 1.

### Implementation Process

**Figure 2.**
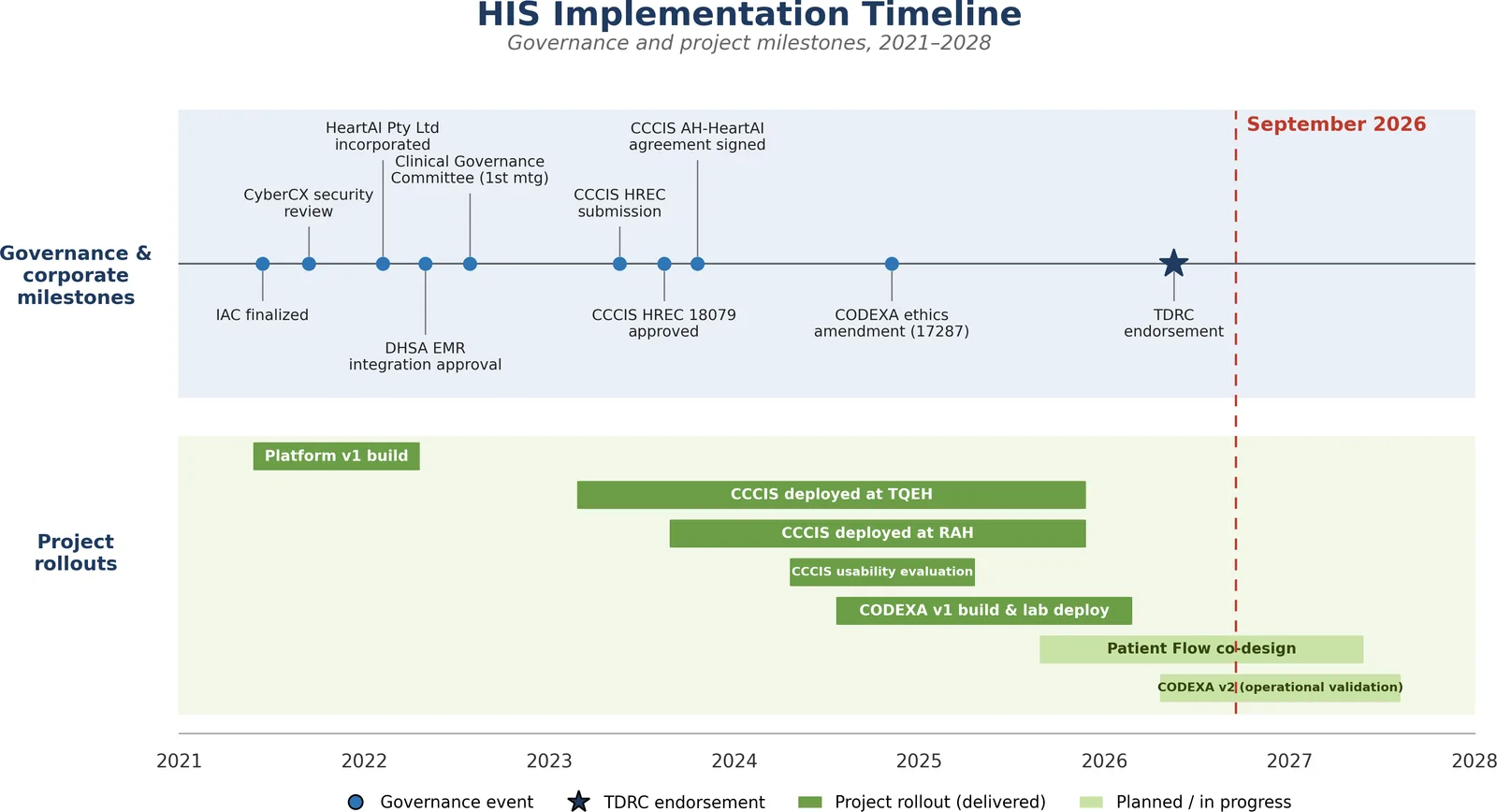
Health informatics system (HIS) implementation timeline. The dashed vertical line indicates work completed up to the writing of this paper (September 2026); paler bars indicate planned or in-progress work. AH: AusHealth; CCCIS: CALHN [Central Adelaide Local Health Network] Critical Care Informatics System; DHSA: Digital Health South Australia; EMR: electronic medical record; HREC: human research ethics committee; IAC: information asset classification; RAH: Royal Adelaide Hospital; TDRC: Technical Design Review Committee; TQEH: The Queen Elizabeth Hospital.

The implementation followed 5 overlapping phases across approximately 5 years (Figure 2): platform development and technical governance (2021‐2022), EMR integration and data validation (2022), clinical application development and pilot deployment (2022‐2024), formal usability evaluation and iterative refinement (2024‐2025), and expansion to additional use cases and user groups (2024-ongoing). Key decisions included a cloud-native rather than on-premises architecture, co-design with end users throughout development, establishment of governance structures before clinical deployment, and phased introduction of applications beginning with the highest-need user group. Decision-making followed a tiered structure, with technical decisions made within parameters set by DHSA, clinical decisions escalated to the Clinical Governance Committee, and strategic decisions requiring approval from CALHN leadership and AusHealth (details in Multimedia Appendix 1).

## Implementation (Results)

### Implementation Status

At the time of writing, the HIS supports 3 applications at explicitly different stages of maturity. CCCIS has been implemented and evaluated. It has been deployed across the 2 CALHN ICUs since 2022 and is in daily clinical use, and a formal usability evaluation has been completed. CODEXA is in operational validation. It is functionally complete and undergoing structured validation with the CALHN clinical coding team, with quantitative model performance reported below; it is not yet in operational use. The Patient Flow application is in co-design with CALHN’s Network Operations Centre and has no deployed components. Table 3 summarizes the status, adoption indicators, and available evaluation metrics for each application.

**Table 3.** Implementation status, adoption indicators, and available evaluation metrics for the 3 HIS applications.

Application	Status and SALIENT stage	Adoption and use indicators	Evaluation metrics available
CCCIS^a^	Implemented and evaluated, stage IV	Deployed across 2 ICUs^b^ (54 beds, ~5000 admissions/year) since 2022; daily use during ward rounds, handovers, and patient reviews; automated ANZICS CORE^c^ registry submission (115 variables); registry staff reliance on CCCIS displays for data collection	SUS^d^ 76/100 (range 65‐95); NASA Task Load Index overall workload 21/100; bespoke questionnaire 7-9/10 for relevance, timeliness, and accuracy (companion publication)
CODEXA	In operational validation, stages II-III	Structured validation sessions with the CALHN^e^ clinical coding team; IHACPA^f^ developer licence secured; not yet in operational use	Production candidate trained on 500,000 episodes, tested on 50,000 held-out episodes: pooled *F*_1_-score 71.0%; nondialysis *F*_1_-score 57.86% (precision 64.72%; recall 55.53%); exact primary diagnosis accuracy 52.17%; exact primary procedure accuracy 53.23% (internal validation report, August 2026)
Patient Flow	In co-design, stage I	Co-design with Network Operations Centre leadership; information-finding interviews and user interface prototype evaluations completed (July 2026); no deployed components	None yet; evaluation planned in line with SALIENT staging

a CCCIS: CALHN (Central Adelaide Local Health Network) Critical Care Informatics System.

b ICU: intensive care unit.

c ANZICS CORE: Australian and New Zealand Intensive Care Society Centre for Outcome and Resource Evaluation.

d SUS: System Usability Scale.

e CALHN: Central Adelaide Local Health Network.

f IHACPA: Independent Health and Aged Care Pricing Authority, the Australian federal body that sets the national efficient price used in activity-based hospital funding.

### Governance Milestones

Ten governance milestones were achieved between 2021 and 2026, from information asset classification (2021) through EMR integration approval and Clinical Governance Committee establishment (May 2022) to TDRC endorsement of the technical design baseline (May 2026) (Figure 2 and Table 1). The sequence reflects the prerequisite relationship between technical governance (platform readiness), corporate governance (formal partnerships), and ethical governance (research activity).

### CCCIS: Implemented and Evaluated

**Figure 3.**
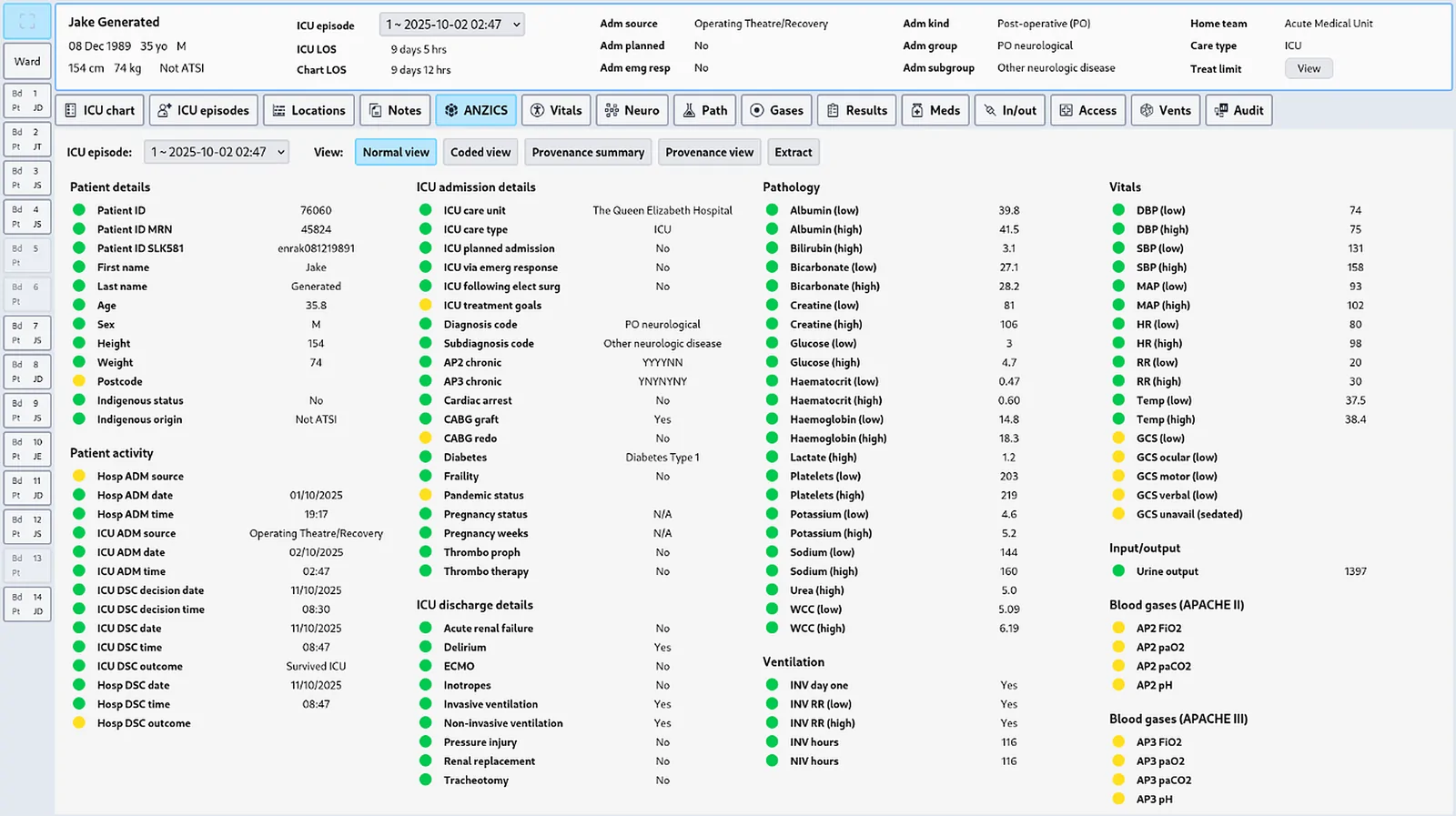
CALHN (Central Adelaide Local Health Network) Critical Care Informatics System (CCCIS) demonstrating the Australian and New Zealand Intensive Care Society Centre for Outcome and Resource Evaluation (ANZICS CORE) data extract using simulated patient data.

CCCIS extracts live data from the Sunrise EMR back end and presents these data through a structured tabular interface modeled on the A0 paper chart that was used in ICUs for over 25 years before the EMR rollout, displaying clinical notes, vital signs, pathology, fluid balance, and ventilation parameters (Figure 3). It is deployed across CALHN’s 2 ICUs (54 beds, approximately 5000 admissions per year) and operates as a research-only, read-only system alongside EMRs, avoiding disruption to existing clinical workflows.

Three indicators of adoption and workflow integration are available. First, clinicians can access CCCIS on clinical workstations during ward rounds, handovers, and ad hoc patient reviews in both ICUs (evaluations ongoing). Second, the system automates real-time submission of Australian and New Zealand Intensive Care Society Centre for Outcome and Resource Evaluation (ANZICS CORE) registry data, covering 115 variables across approximately 5000 ICU admissions annually, replacing a previously manual process [13]. Third, ICU data registry nurses came to use the CCCIS displays as a primary source of clinical information during registry data collection. This reliance on a research-phase system required governance attention, described in the Discussion section, and is direct evidence of integration into daily clinical work.

In a formal usability evaluation, 8 senior CALHN intensivists interacted with CCCIS while it was connected to real patient data, using cognitive walkthrough and think-aloud methods with standardized instruments [14,15]. The mean System Usability Scale score was 76/100 (range 65‐95), in the “good” band on the Bangor adjective scale. Overall NASA Task Load Index workload was low (21/100), with a mental demand score of 38/100 and a frustration score of 18/100. Participants rated the interface at 7-9/10 for clinical relevance, timeliness, and accuracy on a bespoke questionnaire, and the qualitative analysis identified 3 recurrent design principles: structured tabular display, consistency with ICU workflow, and rapid synthesis of physiological, intervention, and trajectory data. Full methods and qualitative findings are reported in a companion publication.

### CODEXA: In Operational Validation

**Figure 4.**
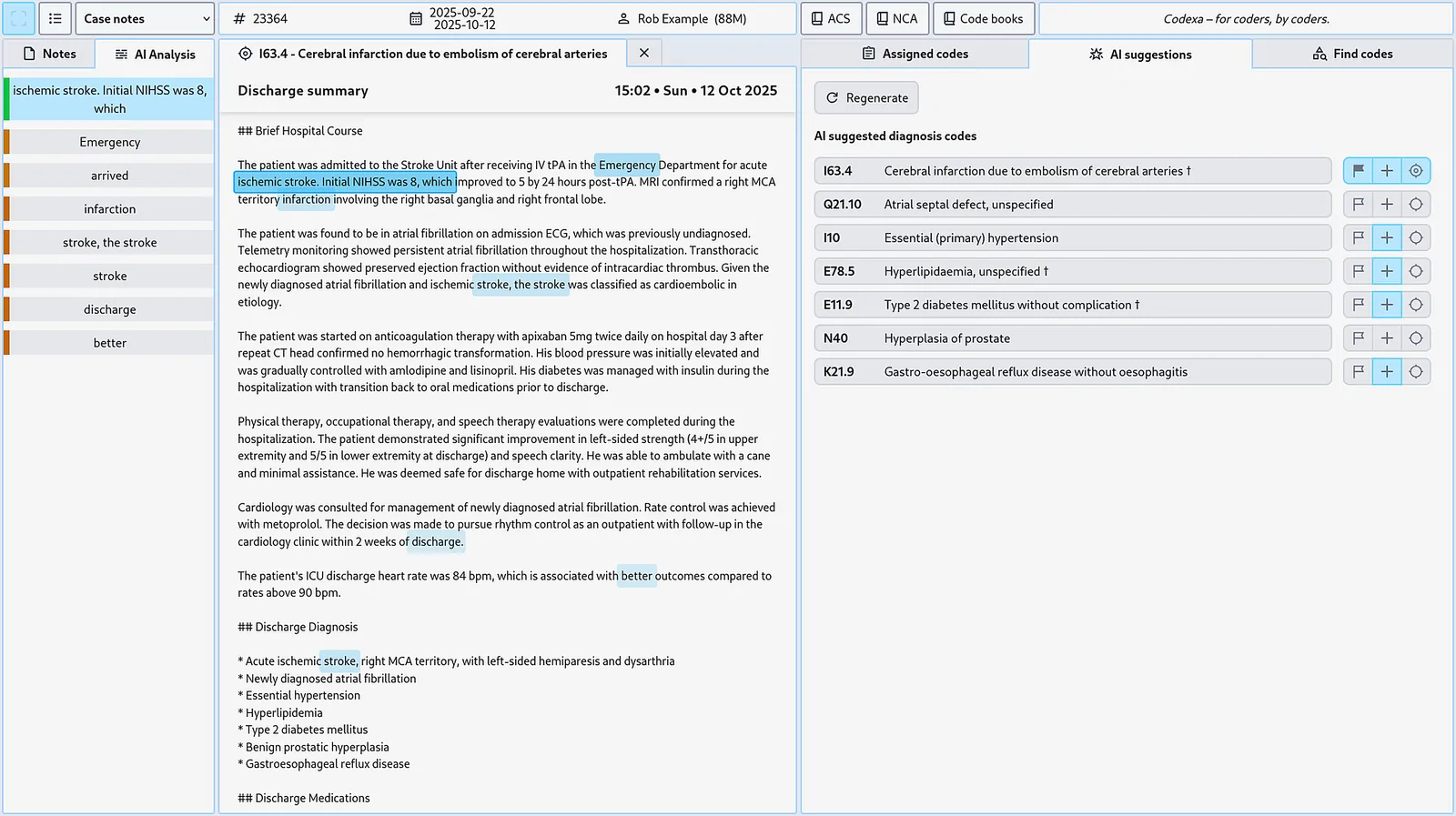
CODEXA demonstrated using simulated patient data (codes are representative and used here to demonstrate layout and process).

CODEXA supports clinical coders who translate patient data into Australian Refined Diagnosis-Related Group classifications for activity-based funding [16]. It extracts EMR data and uses Longformer-based models [17] to associate *ICD-10-AM* (*International Statistical Classification of Diseases and Related Health Problems, Tenth Revision, Australian Modification*) and *Australian Classification of Health Interventions* (ACHI) codes with a patient encounter through an interactive interface that provides weighted summaries and automated cost determinations via national weighted activity unit calculators (Figure 4).

Quantitative validation metrics are available from an internal validation report (August 2026). Successive models were trained on 25,000 ICU episodes, 200,000 episodes, and 500,000 episodes encompassing all admitted services across CALHN, with performance assessed against a held-out test set of 50,000 episodes that were unseen by any model. The current production candidate achieved a pooled *F*_1_-score of 71% across the full hospital case mix. Excluding high-volume, autocoded renal dialysis episodes, which inflate aggregate performance, the model achieved an *F*_1_-score of 57.86% across approximately 32,000 complex acute episodes, with a precision of 64.72%, a recall of 55.53%, an exact primary diagnosis accuracy of 52.17%, and an exact primary procedure accuracy of 53.23%. Performance was strongest in structured, high-volume settings (*F*_1_-scores of 99.4%‐99.7% in dialysis units and 78.7%‐86.9% in focused specialties such as neurology) and weakest in psychiatric and complex multimorbid care (*F*_1_-score of 24%‐47%).

For context, purpose-built coding models, such as pretrained language models for international classification of diseases and multiple synonyms matching networks, achieve *F*_1_-scores of 58% to 61% when evaluated across the full code set on the Medical Information Mart for Intensive Care (MIMIC) benchmarks MIMIC-III and MIMIC-IV [18-20]. Direct comparison should be treated cautiously: the MIMIC benchmarks use curated research datasets and US coding systems, whereas CODEXA was evaluated on uncurated operational EMR data using the Australian *ICD-10-AM*/*ACHI* system. With that qualification, these results suggest that performance is broadly in line with published models under harder, real-world conditions. These internal metrics have not yet been externally peer reviewed; a full evaluation will be reported in a dedicated publication.

### Patient Flow: In Co-Design

The Patient Flow application will give CALHN’s Network Operations Centre near–real-time visibility of patient movement, capacity, and service pressures across the continuum from ambulance arrival to discharge, drawing live EMR data through the HIS. The project is in co-design with Network Operations Centre leadership; information-finding interviews and user interface prototype evaluations with operational staff were completed in July 2026, and no components have been deployed.

### Quantified Outcomes to Date

Definitive clinical impact metrics, including length of stay, mortality, coding accuracy uplift in production use, and patient flow gains, require multiyear deployment data and are not yet available. Available adoption indicators and evaluation metrics are summarized in Table 3, staged according to the SALIENT framework [21].

## Discussion

### Principal Findings

This report describes the development, governance, and deployment of a cloud-native, locally developed informatics platform integrated with a statewide EMR system in a complex public health environment. The implementation required concurrent development of technical infrastructure, multilayered governance, a public-private commercial model, and sustained co-design with clinical end users. Its products sit at 3 deliberately distinct stages of maturity: an implemented and evaluated critical care application in daily use, a coding application in operational validation with quantitative performance now benchmarked against published models, and an operational flow application in co-design.

The HIS shares conceptual foundations with AWARE [5,6], although AWARE was developed in a single, well-resourced academic center and has been evaluated in randomized crossover trials, whereas as of September 2026, evaluation of the SA HIS has addressed usability and model validation rather than clinical outcomes. The usability problems reported in a Finnish national survey of ICU clinical information systems [22] parallel those that this implementation set out to address, suggesting that these are systemic issues across health systems rather than unique to this context.

### The Public-Private Implementation Model

Implementation models for clinical informatics in public hospitals fall into 3 categories: in-house development, procurement from a commercial vendor, and dedicated public-private partnership. In-house development offers maximum institutional control but typically struggles with technical depth, senior engineering recruitment, and the iterative agility that contemporary AI development requires. Vendor procurement provides mature, supported products with clear contractual boundaries but constrains customization for local workflows, introduces ongoing licensing costs, and embeds vendor priorities that may not match public-sector goals.

The model adopted here produced specific realized benefits: rapid iteration through colocated clinical and technical teams, retention of intellectual property in state-led ownership structures, sustained consistency between commercial viability and clinical relevance, and risk-tolerant capital flow through targeted innovation grants under state technical governance. Realized risks include the conflict-of-interest management described in the Methods section, key-person dependency requiring deliberate succession planning, and the governance overhead of operating across 3 institutional bodies. Replication elsewhere depends on the maturity of public-sector innovation funding in the jurisdiction, regulatory clarity around clinician equity in publicly funded health services, and the availability of cofounders able to operate credibly across the clinical, research, operational, and technical domains. Where any of these preconditions are absent, vendor procurement or in-house development may be the more pragmatic route.

### Enablers, Barriers, and Unintended Consequences

Enablers included the practical support of the DHSA technical team, strong executive sponsorship from CALHN leadership, and early collaboration with frontline clinicians, which grounded the mapping of clinical data entry processes to extractable database structures. Barriers included political resistance from stakeholders outside the immediate project team, managed through open communication with data governance authorities and regular demonstrations of the system’s performance and safety; the significant technical difficulty of secondary extraction from an EMR system whose data structures were not designed for it; and milestone-based grant funding that at times reduced capacity and forced difficult prioritization.

One unintended consequence warrants emphasis. Although the HIS remained in a research posture with safeguards against the exposure of clinical workflows to system failures, ICU data registry nurses developed a de facto operational dependency on the CCCIS displays because the system met information needs that the native EMR interface did not. The dependency was identified through governance monitoring and raised concerns about validation status, infrastructure redundancy, and liability. It was managed in 2025 through targeted adjustments to the clinical, ethical, and operational governance of the HIS within the ICU environment. Successful research tools may create operational dependencies before formal transition to operational status, and implementers should plan for this possibility.

### Limitations

This report has 5 limitations. First, it describes a single health network in a single-state health system, limiting direct generalizability to contexts with different governance structures, EMR systems, or organizational cultures. Second, evidence of clinical impact is limited to usability and acceptability data for CCCIS and internal validation metrics for CODEXA; there are no controlled comparisons, clinical outcome data, or health economic analyses, and the CODEXA metrics have not yet been externally peer reviewed. Third, all authors except one hold equity in HeartAI Pty Ltd, creating potential for bias in the reporting of implementation successes and challenges despite the governance mechanisms described. Fourth, the implementation has depended heavily on a small number of key individuals, particularly for technical development, which creates sustainability risk. Fifth, the extraction and integration approaches described are specific to the Sunrise EMR system and may not transfer to other platforms without significant adaptation.

### Lessons for Future Implementers

Three transferable lessons stand out. The first is to establish governance structures early and let them mature with the workload; governance here grew from an early special interest group into the Clinical Governance Committee as deployment expanded. The second is to begin with a constrained task built on a highly validated reference dataset; in this case, the ANZICS CORE registry was used. The third is to plan upstream education for clinical data-entry staff, because errors at the point of entry propagate through every subsequent layer of analysis.

Two structural choices would change with hindsight. A cocommercial lead arrangement retained by HeartAI alongside the funding partner would have made engagement with third parties more efficient as the products matured. Incorporation was delayed until 2022 while public ownership models were exhausted; the process was challenging but ultimately legitimized the decision to incorporate. More broadly, EMR deployment alone is insufficient to realize the value of digitized health data, and complementary platforms that extract, restructure, and present EMR data for specific clinical, operational, and research purposes appear to be a necessary next step in the digital health maturity of large health systems.

### Future Directions

Future work has 3 priorities: completing CODEXA’s operational validation and evaluating its effect on coding accuracy and hospital funding in production use, progressing the Patient Flow application from co-design to deployment and evaluation, and transitioning CCCIS from research to operational status, with the infrastructure and governance changes required by the transition, ahead of wider scaling across SA Health.

## Supplementary material

10.2196/86564 Multimedia Appendix 1 Extended technical, governance, funding, and implementation details.

10.2196/86564 Checklist 1 iCHECK-DH checklist.

## Abbreviations

ACHI: Australian Classification of Health Interventions
ANZICS CORE: Australian and New Zealand Intensive Care Society Centre for Outcome and Resource Evaluation
ASIC: Australian Securities and Investments Commission
AWARE: Ambient Warning and Response Evaluation
CALHN: Central Adelaide Local Health Network
CCCIS: CALHN Critical Care Informatics System
DHSA: Digital Health South Australia
EMR: electronic medical record
HIS: health informatics system
HREC: human research ethics committee
IAC: information asset classification
ICD-10-AM: International Statistical Classification of Diseases and Related Health Problems, Tenth Revision, Australian Modification
iCHECK-DH: Guidelines and Checklist for the Reporting on Digital Health Implementations
ICU: intensive care unit
IHACPA: Independent Health and Aged Care Pricing Authority
IP: intellectual property
MIMIC: Medical Information Mart for Intensive Care
MVP: minimum viable product
SA: South Australia
SIA: security impact assessment
TDRC: Technical Design Review Committee
