# Supplementary material for: A Health Informatics System in the South Australian Public Health Network: Implementation Report
Source: JMIR Med Inform. 2026 Sep 1;14:e86564. doi: 10.2196/86564 (PMC13533040; doi:10.2196/86564)
Supplement: Supplementary file 1 [file medinform-v14-e86564-s001.docx]

**EXTENDED TECHNICAL, GOVERNANCE, FUNDING, AND IMPLEMENTATION DETAIL**

This appendix retains, in full, material condensed from the main text of the manuscript in response to the Round 3 editorial request to reduce overall length. Section numbering follows the order of the main text.

**A2.1 Similar Interventions: Extended Comparison**

Other relevant implementations include national ICU information systems such as those described in Finland, where a survey of ICU clinical information systems reported usability challenges including interface design limitations, customisability constraints, and information retrieval inefficiencies. These findings indicate the widespread nature of the problem the HIS seeks to address. Commercial clinical information systems such as MetaVision (iMDsoft) and Centricity Critical Care (GE Healthcare) offer proprietary solutions, but are typically closed-architecture platforms that limit local customisation and secondary data use. The approach taken in this implementation, a locally developed, open-architecture platform integrated with an existing EMR, prioritises flexibility, co-design, and institutional control over data and intellectual property.

**A2.2 Extended Technical Design and Interoperability**

Resources deployed in Azure are managed with a version-controlled, infrastructure-as-code approach via HashiCorp Terraform. Compute, storage, and networking components are deployed into subnets that separate infrastructure by use-case, encapsulated in a private virtual network. An Azure Red Hat OpenShift cluster provides container orchestration for the HIS's application, service, and analytical workloads. Applications deployed in the cluster are managed via ArgoCD with a GitOps approach.

Services implement Command Query Responsibility Segregation (CQRS), a software pattern that separates data reading operations from data writing operations to improve performance, alongside event-sourcing and Apache Kafka for reactive, high-throughput data flows. These technical choices enable the HIS to process EMR data in near-real-time, which is essential for clinical displays that must reflect current patient status. Applications are developed in Scala.js using Laminar, Airstream, and D3.js, providing performant, type-safe interfaces.

AI models are trained on GPU-enabled hardware in a restricted subnet that only allows connections to the necessary MLOps infrastructure and training data. All artifacts from training are logged and archived securely. Access to models, metrics, or their deployment is restricted based on network and group-based access controls. The ongoing roadmap prioritises federated identity, allowing users to authenticate once across multiple systems, and adaptive analytical orchestration.

Confidentiality is ensured by restricting information, enforcing encryption, limiting user access, and providing secure removal capabilities. Integrity is maintained through auditing modifications, non-repudiation guarantees, unusual access pattern detection, and data recoverability. Personnel security is also managed, with all project team members adhering to prudent digital hygiene and the principle of least-necessary permissions. The system maintains compliance with all relevant laws and policies, particularly those of the SA Government and its health organisations. For sensitive health system information, users must be appropriately credentialed with approved ethics and scope of access.

**A2.3 Extended Governance Detail**

***Technical governance***

Two mandatory evaluations were required to authorise HIS connection to the Sunrise EMR. The first was the Security Impact Assessment (SIA), a policy-driven, ongoing process mandated by the SA Health Information Security Policy Directive. The SIA assesses the HIS's impact on information security and privacy, confirming that it is secure and fit for deployment (initiated in 2021 and ongoing). The second was the Information Asset Classification (IAC), which ensures that digital platforms within the SA Health network are identifiable, matched with business functions, classified by confidentiality, integrity, and availability risks, and operate within compliant, auditable systems. The IAC was completed in 2021. In addition, the HIS underwent an independent review by an external cyber security team, which confirmed that patient data would remain secure and that governance structures met SA Health's standards for clinical deployment.

On 6 May 2026 the Digital Health SA Technical Design Review Committee (TDRC) unconditionally endorsed the HIS platform deployment. TDRC endorsement is the highest formal step in SA Health technical governance and reviews the cumulative output of preceding processes including the SIA, IAC, independent cyber security review, and EMR integration approval. The endorsement followed a 5-year process from initial security review in 2021 to final design baseline approval in 2026. Any subsequent changes to the technical design baseline (for example, new interfacing) will be treated as scope changes and subject to further TDRC review.

***Clinical governance***

To oversee clinical implementation within CALHN, a Clinical Governance Committee was established. Membership includes the CALHN Chief Executive Officer (or delegate), Executive Lead for Critical Care and Perioperative Medicine, Director of Digital Strategy, a consumer representative, and senior representatives from the critical care medical and research teams. Meeting quarterly since May 2022, the committee addresses commercial agreements, ethics, funding and budget management, information security, technical progress, milestones, conflict of interest management, consumer engagement, and data access governance. As additional projects (CODEXA, Patient Flow) have commenced, dedicated subcommittees have been formed to provide focused oversight, reporting into this central governance body.

***Corporate governance***

To enable formal, legally binding relationships with public institutions and funders, HeartAI Pty Ltd was incorporated in February 2022. The corporate structure enabled partnerships with SA Health, Digital Health SA, CALHN, and AusHealth. Upon incorporation, equity was allocated to inaugural shareholders in recognition of prior work contributing to the informatics system's development, including early technical builds, software architecture, and the intellectual property underlying its clinical, technical, and operational design. This model provides accountability, clarity of ownership, and the capacity to engage in commercial agreements while maintaining consistency with public sector objectives, including the declaration and management of actual or perceived conflicts via SA Health policies.

***Ethical governance***

Ethics approval for the CALHN Critical Care Informatics System (CCCIS) project was granted by the CALHN Human Research Ethics Committee in 2023 (Reference No. 18079). The project was deemed low to negligible risk, with a waiver of patient consent approved for participating hospitals. This ethics approval pertains to the research activities conducted using the HIS (data collection, usability evaluation, outcome assessment), and does not cover the HIS's technical development, which preceded the formal research programme and was governed by the technical governance processes described above. The distinction between infrastructure development (TDRC) and research activity (HREC) reflects the sequential nature of the implementation process. Subsequent amendments to the master CCCIS ethics protocol extended coverage to include CODEXA (Reference No. 17287) and the Patient Flow project (pending HREC review).

**A2.4 Extended Funding and Commercial Detail**

***Research grants***

The initial concept and development of the HIS was conceived by three authors (LD, JM, AP), representing technical, clinical, and corporate domains respectively. Early platform builds, implementation frameworks, and project protocols were completed on personal time, with the lead technical architect (LD) transitioning to full-time unpaid development prior to the incorporation of HeartAI Pty Ltd. In December 2022, the company received its first research grant, an internal CALHN Innovation Award of AU$200,000 over 2 years, to support the CCCIS project. A second grant followed in June 2023, with AU$312,000 over 2 years awarded by AusHealth under a Collaborative Research and Commercialisation Agreement. AusHealth serves both as commercialisation partner and as a self-funded medical research charity that reinvests profits into health innovation. Further funding included AU$1,130,000 over 3 years (May 2024) for the CODEXA project, wholly funded and owned by AusHealth, and AU$200,000 over 1 year (April 2026) for the Patient Flow project, jointly funded and owned by CALHN, AusHealth, and HeartAI Pty Ltd (a similar agreement was used for CCCIS).

***Private equity***

Following incorporation, HeartAI Pty Ltd attracted intermittent private investment and in-kind contributions from its founding team and early collaborators. As of early 2026, 11 equity holders are recorded in the company's shareholder registry, available via the Australian Securities and Investments Commission (ASIC). The total value of private equity contributions is not disclosed in this report due to commercial confidentiality requirements of a privately held company. The authors confirm that private investment has been directed toward core platform development and corporate operations, and has not influenced the research agenda, which is set by the publicly funded grant agreements with CALHN and AusHealth. Private contributions have been a minority of total project funding relative to the competitive research grants described above. The hybrid funding model has enabled sustained technical development while maintaining independence and consistency with the public health system's strategic priorities.

***Commercial agreements***

The for-purpose model integrates the agility of a private company with the infrastructure, governance, and public value mandate of the health system. Under the agreement, CCCIS is jointly owned by CALHN and AusHealth, so the South Australian public remains a direct beneficiary. For CODEXA, AusHealth retains product ownership, with a share of revenue directed to HeartAI Pty Ltd to support ongoing viability and reinvestment in medical research. The Patient Flow project, developed using background IP, falls under the same commercial framework as CCCIS. This model will be replicated if and when the informatics system scales across CALHN and SA Health more broadly.

***Sustainability***

Long-term sustainability was designed around an interdependent set of capabilities rather than a single funding source. An agile yet secure governance framework balances adaptability with privacy, safety, and equity, drawing on insights from AI governance pilots. The for-purpose commercial model couples public-sector accountability with risk-tolerant capital flow guided by ethical principles for AI in healthcare. Dedicated intellectual property and legal expertise has supported collaboration in a space where AI-related IP frameworks remain nascent. Formal and informal stakeholder forums have kept development problem-driven and user-centred. Beyond the current grant-funded phase, the roadmap includes transition from research-only to operational funding through CALHN's digital health budget; progressive FHIR adoption to reduce vendor dependency; workforce development for in-house platform maintenance; and shared-service models that would allow other SA Health Local Health Networks to adopt HIS components without duplicating development costs.

**A2.5 Extended Implementation Process Detail**

Workflow integration for CCCIS involved deploying the application as a web-based tool accessible alongside the existing EMR on clinical workstations in CALHN's two ICUs. CCCIS operates as an information delivery system; it reads data from the EMR and does not write back to it, avoiding disruption to existing clinical workflows. Clinicians access CCCIS through a web browser during ward rounds, handovers, and ad hoc patient reviews. Feedback from initial users was collected through informal channels and structured usability evaluation sessions, with findings used to drive iterative interface refinements.

Governance pathways for decision-making followed a tiered structure: technical decisions were made by the development team within the parameters set by DHSA; clinical decisions were escalated to the Clinical Governance Committee; and strategic decisions regarding funding, commercialisation, and expansion required approval from both CALHN leadership and AusHealth. This tiered approach balanced agility in day-to-day development with accountability for decisions affecting clinical care and institutional resources.
