# Supplementary material for: A Health Informatics System in the South Australian Public Health Network: Implementation Report
Source: JMIR Med Inform. 2026 Sep 1;14:e86564. doi: 10.2196/86564 (PMC13533040; doi:10.2196/86564)
Supplement: Supplementary file 2 [file medinform-v14-e86564-s002.docx]

**Appendix 1. iCHECK-DH Reporting Checklist**

*Manuscript #86564 — A Health Informatics System in The South Australian Public Health Network: An Implementation Report*

This manuscript reports a digital health implementation following the iCHECK-DH framework (Perrin Franck et al., JMIR 2023; doi:10.2196/46694). The table below maps each iCHECK-DH reporting item to the corresponding section of the main manuscript. Where the framework lists an item that does not apply to this implementation, this is noted explicitly.

| **Domain** | **Item** | **Description** | **Reported in** |
| --- | --- | --- | --- |
| Title and Abstract | 1 | Title identifies the report as a digital health implementation | Title (front matter) |
| Title and Abstract | 2 | Structured abstract with Background, Objectives, Methods, Results, Conclusions | Abstract (front matter) |
| Introduction | 3 | Context: description of the broader setting in which the implementation took place | Introduction: Context and Problem |
| Introduction | 4 | The Problem: specific problem the digital health intervention addresses | Introduction: Context and Problem |
| Introduction | 5 | The Intervention: description of the digital health intervention deployed | Introduction: The Intervention |
| Introduction | 6 | Similar Interventions: description of related digital health implementations | Introduction: Similar Interventions; Multimedia Appendix 2 (A2.1) |
| Methods | 7 | Aims: specific aims of the implementation | Methods: Aims, Objectives, and Evaluation Framework |
| Methods | 8 | Objectives and evaluation framework: stated objectives with measurable indicators | Methods: Aims, Objectives, and Evaluation Framework; Table 1 |
| Methods | 9 | Blueprint summary: technical and architectural description of the intervention | Methods: Platform Architecture and Data Governance; Figure 3; Multimedia Appendix 2 (A2.2) |
| Methods | 10 | Participating entities: stakeholders and their roles in the implementation | Methods: Governance; Multimedia Appendix 2 (A2.3) |
| Methods | 11 | Sustainability: strategy for long-term continuation of the intervention | Methods: Funding and Commercial Model; Multimedia Appendix 2 (A2.4) |
| Methods | 12 | Budget planning: financial resources required and how they were secured | Methods: Funding and Commercial Model; Table 2; Multimedia Appendix 2 (A2.4) |
| Methods | 13 | Implementation process: chronological description of how the intervention was deployed | Methods: Implementation Process; Figure 4; Multimedia Appendix 2 (A2.5) |
| Results | 14 | Governance milestones: governance approvals achieved during implementation | Results: Governance Milestones; Figure 4 |
| Results | 15 | Coverage: extent of the implementation (population, sites, services covered) | Results: Implementation Status |
| Results | 16 | Outcomes: impact and effectiveness of the digital health implementation | Results: CCCIS, CODEXA, and Patient Flow subsections; Quantified Outcomes to Date; Table 3 |
| Discussion | 17 | Principal findings: summary of main findings of the implementation | Discussion: Principal Findings |
| Discussion | 18 | Comparison with prior work: how this implementation compares to similar efforts | Discussion: Principal Findings; The Public-Private Implementation Model |
| Discussion | 19 | Implementation enablers and barriers: factors that facilitated or hindered the implementation | Discussion: Enablers, Barriers, and Unintended Consequences |
| Discussion | 20 | Unintended consequences: unexpected outcomes (positive or negative) of the implementation | Discussion: Enablers, Barriers, and Unintended Consequences |
| Discussion | 21 | Limitations: limitations of the implementation and of the report | Discussion: Limitations |
| Discussion | 22 | Implications for practice and generalisability | Discussion: Lessons for Future Implementers |
| Discussion | 23 | Future directions: planned next steps for the implementation | Discussion: Future Directions |
| Other Information | 24 | Regulatory approvals (if applicable) | Regulatory Approvals; Methods: Governance (ethical governance) |
| Other Information | 25 | Conflicts of interest | Conflicts of Interest; Methods: Managing Conflicts of Interest |
| Other Information | 26 | Funding sources | Methods: Funding and Commercial Model; Table 2 |
| Other Information | 27 | Authors' contributions | Authors' contributions (front matter) |

*Note. The iCHECK-DH framework is described in: Perrin Franck C, et al. iCHECK-DH: guidelines and checklist for the reporting on digital health implementations. JMIR Med Inform. 2023. doi:10.2196/46694.*
